# Review and perspectives of enhanced volatile fatty acids production from acidogenic fermentation of lignocellulosic biomass wastes

**DOI:** 10.1186/s40643-021-00420-3

**Published:** 2021-08-02

**Authors:** Jiachen Sun, Le Zhang, Kai-Chee Loh

**Affiliations:** 1 Department of Chemical and Biomolecular Engineering, National University of Singapore, 4 Engineering Drive 4, Singapore, 117576 Singapore; 2 NUS Environmental Research Institute, National University of Singapore, 1 Create Way, Create Tower #15-02, Singapore, 138602 Singapore; 3 Energy and Environmental Sustainability for Megacities (E2S2) Phase II, Campus for Research Excellence and Technological Enterprise (CREATE), 1 CREATE Way, Singapore, 138602 Singapore

**Keywords:** Volatile fatty acids, Acidogenic fermentation, Enhancing strategies, Lignocellulosic biomass wastes, Resource recovery

## Abstract

Lignocellulosic biomass wastes are abundant resources that are usually valorized for methane-rich biogas via anaerobic digestion. Conversion of lignocellulose into volatile fatty acids (VFA) rather than biogas is attracting attention due to the higher value-added products that come with VFA utilization. This review consolidated the latest studies associated with characteristics of lignocellulosic biomass, the effects of process parameters during acidogenic fermentation, and the intensification strategies to accumulate more VFA. The differences between anaerobic digestion technology and acidogenic fermentation technology were discussed. Performance-enhancing strategies surveyed included (1) alkaline fermentation; (2) co-digestion and high solid-state fermentation; (3) pretreatments; (4) use of high loading rate and short retention time; (5) integration with electrochemical technology, and (6) adoption of membrane bioreactors. The recommended operations include: mesophilic temperature (thermophilic for high loading rate fermentation), C/N ratio (20–40), OLR (< 12 g volatile solids (VS)/(L·d)), and the maximum HRT (8–12 days), alkaline fermentation, membrane technology or electrodialysis recovery. Lastly, perspectives were put into place based on critical analysis on status of acidogenic fermentation of lignocellulosic biomass wastes for VFA production.

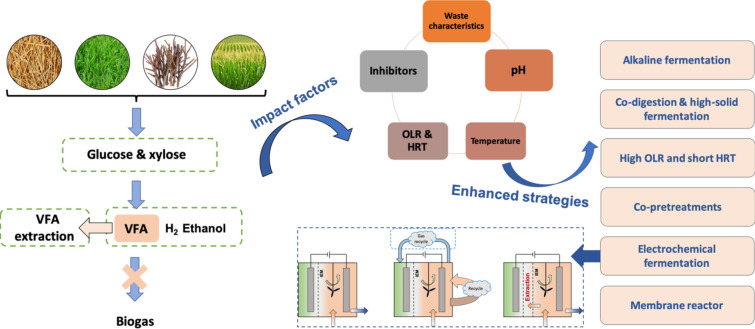

## Highlights

- Strategies to enhance acidogenic fermentation of lignocellulosic biomass presented.
- Optimal parameters for anaerobic digestion and acidogenic fermentation are compared.
- Challenges of electrochemical fermentation of lignocellulosic biomass are discussed.
- Membrane technology and its further optimization are effective for VFAs extraction.

## Introduction

Lignocellulosic biomass wastes, comprising mainly agricultural wastes, forest wastes and grass, are carbon-rich raw materials for that can be exploited for the production of clean biofuels and valuable chemicals. As reported by the World Bioenergy Association, right up to 2016, lignocellulosic biomass wastes accounted for 97% of biomass supply, with the remaining 3% made up by other organic wastes, such as food waste and waste sludge (World Bioenergy Association 2016). The same report also projected that the amount of agricultural and forest wastes is slated to increase significantly by 1040% and 60%, respectively, by 2035. In face of such abundant unexploited biomass, it is therefore essential for innovative technologies be developed to provide valorization of this biomass to unlock their potential bioenergy and bioresource.

Anaerobic digestion (AD) has been recognized as an effective technology to convert organic wastes into biomethane (Zhang et al. 2019). Generally, AD consists of four stages, namely, hydrolysis, acidogenesis, acetogenesis, and methanogenesis (Tezel et al. 2011). Under suitable operating conditions, for example a retention time of 20–30 days (Mao et al. 2015), AD can proceed to completion and the targeted product (biomethane) is produced and utilized as fuels. On the other hand, if the AD process were interrupted, for example before methanogenesis, many intermediate compounds, like volatile fatty acids (VFA), hydrogen, and ethanol can be generated. VFA are regarded as a more valuable product than biomethane as they can be further converted into high value-added chemicals (e.g., bioplastic) and biofuels (e.g., biodiesel) (Kim et al. 2013; Tu et al. 2019; Zhang et al. 2021). Thus, the burden of a relatively long retention time and desire for more efficient lignocellulose valorization tend to encourage accumulation of more VFA rather than the eventual production of biomethane.

**Fig. 1:**
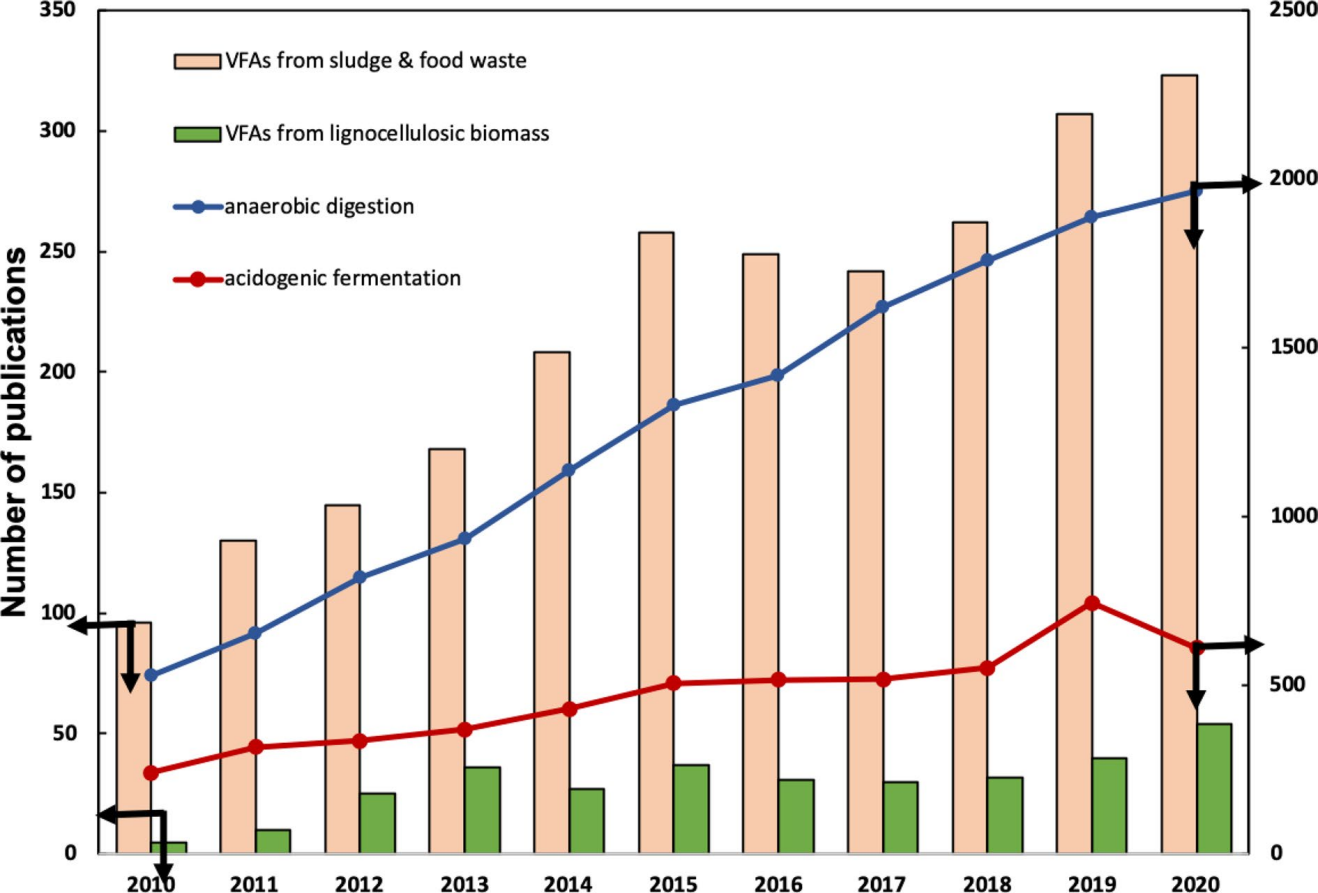
Number of annual publications from 2010 to 2020 on “anaerobic digestion”, “volatile fatty acid OR acidogenic fermentation”, “volatile fatty acid OR acidogenic fermentation AND (food waste OR sludge)” and “volatile fatty acid OR acidogenic fermentation AND lignocellulos*” (based on search results in Scopus database)

Figure 1 shows the annual publications in the scientific literature on VFA production in the past decade. It can be seen that the number of publications on AD for biomethane production is sixfold higher than that of acidogenic fermentation for VFA production. Among the surveyed studies on acidogenic fermentation, half of them utilized sludge and food waste as feedstocks while the studies using lignocellulosic biomass wastes as feedstocks accounted for only 3.7% and 6.3%, compared to the total publications on VFA production and the publications on using sludge and food wastes as feedstocks for VFA production, correspondingly. This phenomenon is likely due to the fact that sludge and food wastes are relatively easier to degrade compared to the more recalcitrant lignocellulosic biomass and that a shorter retention time for acidogenic fermentation also limits the hydrolysis and acidogenesis processes. Based on this, there are significant prospects to investigate conversion technologies for lignocellulosic biomass wastes into VFA instead of generating only biomethane.

In reviewing the literature, it is anticipated that much of what has been done to optimize AD for biomethane production, such as the optimization of parameters and operating conditions, as well as enhancing technologies can also be applied in the studies for VFA production. For example, it is normal to determine the maximum loading rate of feedstocks to avoid acidic inhibition in AD (Zealand et al. 2017), naturally we can stride this threshold for higher VFA accumulation. Of course, the differences in the targeted products will definitely influence the operating parameters (Eryildiz and Lukitawesa 2020; Zhang et al. 2005), product accumulation (Liu et al. 2011; Wainaina et al. 2019a), and product separation approaches (Aydin et al. 2018; Torri et al. 2019). Furthermore, although several previous reviews had reported on the topic of acidogenic fermentation mainly on sludge and food wastes, the current review focuses on promising strategies for sustainable utilization and valorization of lignocellulosic biomass wastes into VFA production, as well as insights on various parameter enhancement strategies against specific characteristics of acidogenic fermentation, such as the use of novel membrane bioreactors for product extraction, and integration with electrochemical pretreatment and in situ bioelectrochemical bioreactor.

This review will start with an analysis of the characteristics of lignocellulosic biomass wastes followed by the typical metabolism pathways of acidogenic fermentation, with the hope of understanding the acidogenic processes from the perspective of the microorganisms involved in the process. Following that, the effects of operating parameters for VFA accumulation are summarized in “Factors influencing VFA production” section, in which the differences between acidogenic fermentation and AD are highlighted, and the parameter optimization strategies are summarized in “Optimization of operating parameters” section. Novel and cost-effective pretreatments are then critically discussed in “Combination of effective pretreatments” section in order to overcome the recalcitrant barriers of lignocellulosic biomass wastes. Finally, some promising technologies are proposed as integration into acidogenic fermentation bioreactors to improve VFA yields and their respective applications are briefly discussed.

## Acidogenic fermentation of lignocellulosic biomass wastes

### Characteristics of lignocellulosic biomass wastes

Lignocellulosic biomass wastes mainly include wood, grass, energy crops, and agricultural and forest residues. Chemically, lignocellulosic biomass wastes comprise cellulose, hemicellulose, and lignin, trace amounts of extractive fractions (e.g., tannins, resins, fatty acid), and inorganic salts (Orfão et al. 1999). Table 1 shows the compositions of some selected lignocellulosic biomass wastes. In general, the cellulose, hemicellulose and lignin contents of lignocellulosic biomass wastes are 30–50 wt%, 15–40 wt%, and 10–30 wt%, respectively. High cellulose, high hemicellulose contents and high C/N ratio are the three main characteristics of lignocellulosic biomass wastes. For the biodegradation of yard waste and horticultural waste, including grass, leaves, and woody twig, previous studies had usually mixed the three kinds of wastes as feedstocks according to the formulation of 65% tree leaves, 33% grass, and 2% woody twigs (Panigrahi et al. 2019; Sharma et al. 2019). The C/N ratio of grass had been in the range of 20–40, while that of woody chips could even reach above 100 (Tahboub et al. 2008). The effect of C/N ratio on acidogenic fermentation of lignocellulosic biomass is discussed in detail in “Waste characteristics” section.

**Table 1:** Compositions of some surveyed lignocellulosic biomass wastes

Substrates	Cellulose^a^	Hemicellulose^a^	Lignin^a^	C/N ratio	References
Wheat straw	39	31	9	44	Liu et al. (2019c)
Corn straw	36	28	5	54	Wang et al. (2018)
Rice straw	38	29	15	43	Kainthola et al. (2019)
Yard waste	18	23	10	60	Zhang et al. (2018)
Grass clipping	42	35	7	–	Wang et al. (2019a)
Wheat husk	19	18	10	50	Sun et al. (2019)
Leaves	30	27	13	37	Yao et al. (2017)
Napier grass	45.7	33.7	20.6	26	Reddy et al. (2012)
Giant reed	51.7	26.6	8.0	54.7	Corneli et al. (2016)
Sunflower stalk	34.0	20.8	29.2	–	Monlau et al. (2012)
Hardwood birch	41.0	30.0	24.6	–	Martínez-Abad et al. (2018)
Softwood spruce	43.0	24.2	28.8	–	Mohsenzadeh et al. (2012)

^a^% on dry weight basis

Within the structure of lignocellulosic biomass, cellulose refers to a linear polymer of β-1,4 glucan, which has the tendency to form intra- and inter-molecular hydrogen bonds through the hydroxyl groups, and aggregate into their crystalline structure (Pu et al. 2008). The strong hydrogen bonds make cellulose highly insoluble in standard solvents (e.g., water) and resistant to enzymatic hydrolysis (Galbe and Zacchi 2012). Hemicellulose is a complex structure of carbohydrates, including mainly xylose, arabinose, glucose, and some acids. Xylose is a dominant hemicellulose component in grass and hardwoods. Some pretreatments like alkali addition can destroy the xylan between cellulose and hemicellulose to accelerate the hydrolysis process (Kim et al. 2016). Unlike cellulose, hemicellulose has a relatively lower degree of polymerization (Saha et al. 2019), and is highly hydrophilic and more amenable to hydrolysis. Lignin is a phenolic polymer and is regarded as the most recalcitrant component of plant cell walls. Generally, softwood has a higher fraction of lignin than hardwood. In addition to cellulose crystallinity, linkage between cellulose and hemicellulose, and cementation and steric hindrance from lignin both increase the difficulty for enzymatic hydrolysis of cellulose (Zhang and Hu 2018). Nevertheless, lignocellulosic biomass, cellulose and hemicellulose are sugars-rich resources that can be harnessed for biofuels productions using anaerobic digestion/fermentation.

### Acidogenic fermentation pathways of lignocellulosic biomass wastes

**Fig. 2:**
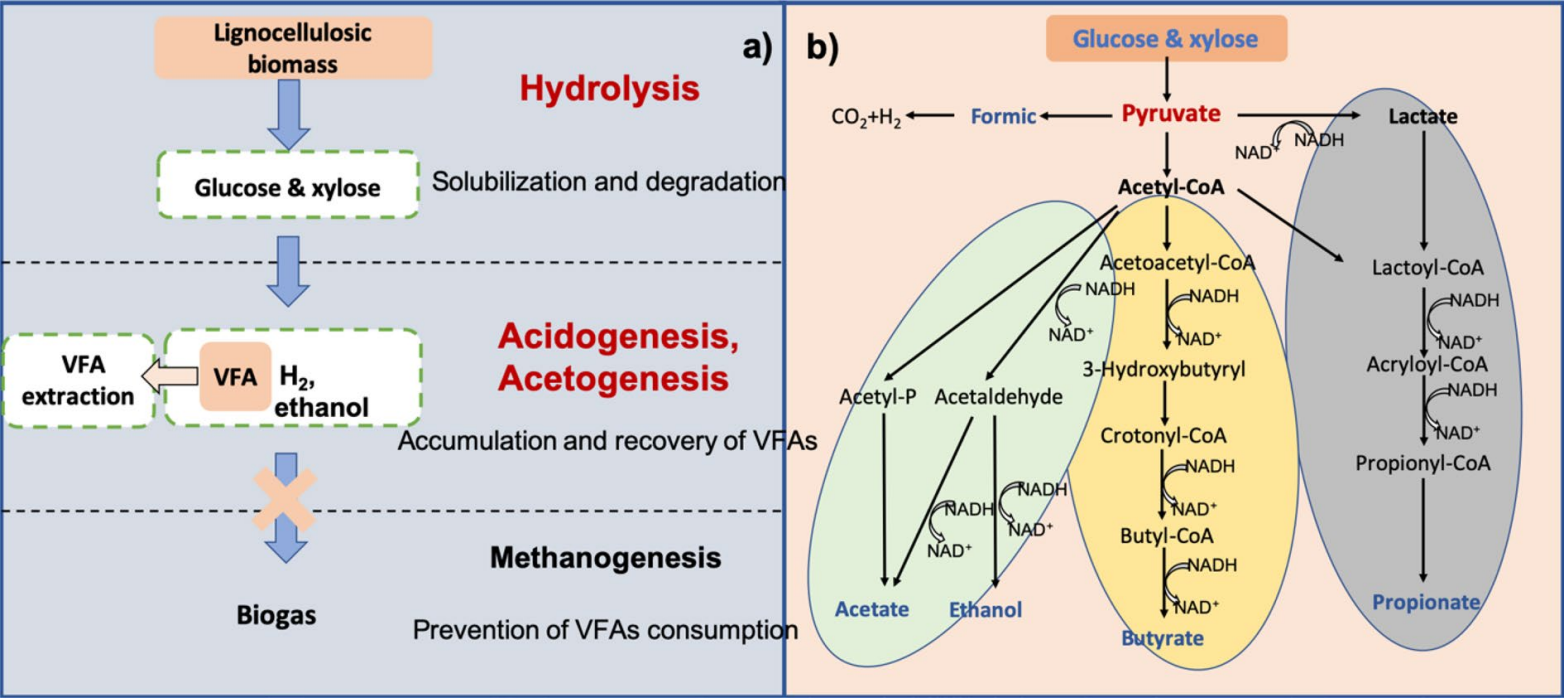
**a** Schematic flow of formation of VFA during anaerobic digestion; **b** typical metabolic pathways of acidogenic fermentation (Adapted from Zhou et al. (2018) with permission/license granted by the publisher)

As previously mentioned, AD consists of four steps, i.e., hydrolysis, acidogenesis, acetogenesis, and methanogenesis, each of which involves the participation of different microorganisms (Fig. 2a). Firstly, lignocellulosic biomass wastes are solubilized and degraded into glucose and xylose during hydrolysis (generally considered the rate-limiting step of AD). In order to enhance access to the various waste characteristics, pretreatments are required to accelerate hydrolysis, and enhance production yields. For example, supplementation with various microbial strains, such as *Bacteroidetes*, *Proteobacteria* and *Firmicutes* phylum contributed to lignocellulose hydrolysis (Sun et al. 2019). Incidentally, the hydrolysis of biomass had been positively correlated to the abundance of *Bacteroidetes* (Regueiro et al. 2012).

Hydrolyzed monosaccharides are then utilized by acidogens and acetogens to generate VFA and ethanol. Acetic, butyric and propionic acids are dominant VFA components from the various lignocellulosic substrates (Mockaitis et al. 2020; Wang et al. 2019a). Acidification pathways can hence be classified into acetate–ethanol type, butyrate-type, and propionate-type, respectively (Fig. 2b). In the network of acidogenic metabolic pathways, pyruvate (CH_3_COCOH) plays a crucial role as it can be converted into a range of products, like acetate, ethanol, propionate, and H_2_ (Zhou et al. 2018). Acetate–ethanol pathway refers to one that generates acetate and ethanol from acetyl-CoA (CH_3_COSCoA) through acetyl-P and acetaldehyde, along with the production of minuscule amounts of CO_2_ and H_2_ (Kandylis et al. 2016). The proportion of acetic acid could account for 40–88% of the total amount of VFA production under different conditions (Lu et al. 2018; Song et al. 2019b), while ethanol contributed only around 10–25% (Song et al. 2019b). Accumulation of ethanol could also be toxic to the microorganisms and cause the loss of carbon sources when more of the carbon molecules got converted to ethanol rather than acids (Zhou et al. 2018). In addition, acetate could also be converted via syntrophic oxidation of ethanol and long chain fatty acids (e.g., butyrate and propionate) at a longer retention time (Zhou et al. 2018). Butyrate-type pathway derives from pyruvate through acetyl-CoA to butyric acid along with CO_2_ and H_2_ (C_6_H_12_O_6_==CH_3_CH_2_CH_2_COOH + 2CO_2_ + 2H_2_) (Liu et al. 2013), where the proportion of butyric acid was about 5–15% (Kim et al. 2013) (Lu et al. 2018; Wang et al. 2019a). Propionate can be produced from both acetyl-CoA and intermediate lactate by *Propionibacterium* spp. and many others (3CH_3_CHOHCOOH==CH_3_COOH + 2CH_3_CH_2_COOH + CO_2_ + H_2_O) (Seeliger et al. 2002), and its content were in the range of 0.2–3 g/L based on the different operation (Lu et al. 2018; Mockaitis et al. 2020; Sawatdeenarunat et al. 2017). The metabolism of the entire system associates with a great deal of enzymes and electron transfers.

pH control has been regarded as an essential operating condition to influence the progression of the fermentation processes. Acidic pH (below pH 6.5) has traditionally been used to operate the bioreactor in continuous operation, while the neutral pH range was usually used to in batch with the help of methanogenesis inhibitors. At an initial low pH of 4–4.5, acetate–ethanol type fermentation is predominant while pH of 4.5 is optimal for maximizing the yield of ethanol (Ren et al. 1997). In terms of the microcosm composition, species *Clostridium* and *Enterobacter* that can produce acids (mainly like acetic, butyric, propionic, and lactic acids) are common in the fermentation reactor fed with lignocellulosic feedstocks (Kumar et al. 2016). The abundance of *Bifidobacterium thermacidophilium* increased at pH 4, producing acetic and lactic acid (Zhu et al. 2003), and *Lactobacillus acidophilus*, which also prefers low pH (below pH 5) producing acids from the sources of sugars (Sanders and Klaenhammer 2001). A pH range of 5–6, tends to facilitate the propionate-type fermentation, and a pH of higher than 6 is therefore suggested for the acidogenic operation in order to avoid a high propionic yield. Genus *Veillonella gazogenes* and *Clostridium propionicum* are two popular species in mixed cultures during propionate-type fermentation (Khanal et al. 2004). Finally, butyrate-type fermentation takes place at a pH lower than 5 and higher than 6, during which genus *Clostridium butyricum* and *Butyrivibrio* function for butyric acid production. The key enzyme controlling butyrate formation was higher in concentration in the cells at pH 6.3 (Zhu and Yang 2004). An increase of propionate production in the range of pH 5–6 also led to the decrease of butyric acid, thus, butyric acid-type fermentation tended to convert to propionate-type fermentation due to the pH variation. Alkaline pH has also been investigated; it has been found that acetate production increased with judicious pH control and yield of butyric acid was highly enhanced at pH 12 (Liu et al. 2012).

## Factors influencing VFA production

**Fig. 3:**
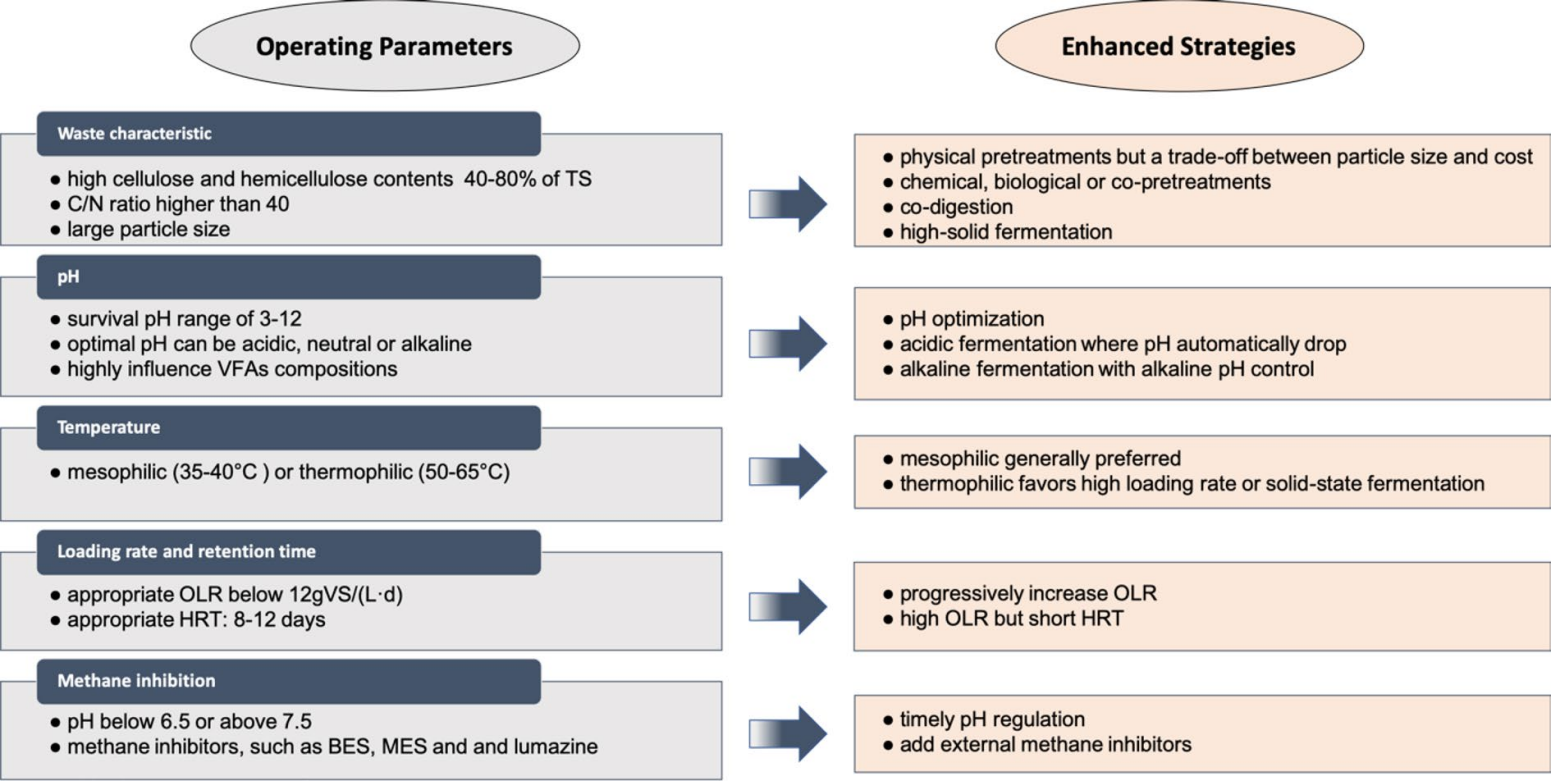
Workflow for optimization of operating parameters during acidogenic fermentation

Figure 3 presents the effects of operating parameters during acidogenic fermentation, including waste characteristics, pH, temperature, organic loading rate, and hydraulic retention time. Methane inhibition and the available enhanced strategies were also involved.

### Waste characteristics

As indicated in “Characteristics of lignocellulosic biomass wastes” section, the total content of cellulose, hemicellulose and lignin accounts for 50–90% of lignocellulosic biomass wastes. As only cellulose and hemicellulose can be converted into biofuels using AD, higher contents of cellulose and hemicellulose means higher potential of VFA yield. Hardwoods possess more cellulose and hemicellulose than softwoods. Table 1 also shows that lignocellulosic biomass wastes usually have a C/N ratio higher than 40. Generally, a C/N ratio of 20–30 has been regarded as an amiable environment, which could balance carbon and nitrogen nutrients necessary for microbial growth (Wang et al. 2012). A low C/N ratio could release excessive ammonia, and the accumulated ammonia would lead to an increase of pH and an unbalance nutrient composition, which were regarded to inhibit the methanogens in AD (Shi et al. 2017). Although acidogens could survive in the moderate alkaline environment, it has been reported that the VFA yield from C/N ratio of 20, 25 and 30 increased 70%, 140% and 160%, correspondingly compared to C/N ratio of 15 (Liu et al. 2008). Conversely, a moderately high C/N ratio could cause the decrease of pH and favor VFA accumulation. Acidogenic fermentation could tolerate high C/N ratio up to 40, VFA yield of which was found to be equivalent to that acting on a C/N ratio of 20 (Liu et al. 2021). It is reasonable to expect that acidogens acting in acidogenic fermentation using most lignocellulosic biomass as feedstocks to be rather adaptable to various C/N ratios. Table 2 shows acidogenic fermentation with various types lignocellulosic biomass wastes and the adopted operating parameters. Generally, pretreatments, operating conditions, and VFA yield are frequently dependent on the type of the lignocellulosic biomass wastes. In particular, when it comes to yard waste and energy crops, the cellulose and hemicellulose contents are also affected by the climate and environment (Song et al. 2019a).

**Table 2:** Acidogenic fermentation from various types of lignocellulosic biomass wastes and the operating parameters

Substrates	Pretreatments	Chemical compositions	Reactor	Operation mode	VFA yields	Product compositions	Main bacterial characteristics	References
Grass clipping	Ultrasound-Ca(OH)_2_	4 and 32 g/g volatile solids (VS) glucose and xylan released	250 mL, 35 °C, 120 rpm	Batch 12d	578 mg/g volatile solids (VS), increase 116%	82% acetic, 4% propionic and 4% butyric acids	Bacteria class: *Clostridia* and *Bacteroidia*	Wang et al. (2019a)
Grass	Carbide slag	Reduced 6% lignin and 8% hemicellulose	250 mL, 35 °C	Batch	220 mg/g volatile solids (VS), increase 38%	78% acetate, 4% propionate and 8% butyrate	*Clostridium, Bacteroides*	Tao et al. (2019)
Napier grass	Milling	–	250 mL flasks, 37 °C, 100 rpm, micro O_2_,	Batch	107.25 mg/g volatile solids (VS)	50–70% acetic and 20–50% propionic acids	–	Sawatdeenarunat et al. (2017)
Japanese cedar	Vibration milling	–	2 L, 39 °C, 30–40 rpm, pH 6.5	Semi-continuous	76 mg/g volatile solids (VS)	37% acetic and 60% propionic acids	*F. succinogenes* (cellulolytic bacterium), *P. ruminicola* (synergistically with cellulolytic bacteria), *S. ruminis* (propionate-producing)	Agematu et al. (2017)
Fallen leaves	Lime pretreatments	–	250-mL flasks with 10% inoculum, 37 °C, pH 8	Batch 5d	1.06 g/g volatile solids (VS), increase 160%	70% acetic and 20% butyric acids	–	Kim et al. (2013)
Sorghum stalks	Milling and acid	Hemicellulose degraded by 60%	135 mL, 55 °C,	Two steps	424 mg/g volatile solids (VS), increase 84% acetate and 116% butyrate	2.17 g/L acetic and 2.07 g/L butyric acids	–	Islam et al. (2018)
Corn stalk	Acid pretreatment	Hemicellulose reduced by 12%, cellulose increased by 15%	500 mL flask, 50 °C, 15 days, pH 7	Batch	150 mg/g volatile solids (VS)	0.86 g/L formic, 0.59 g/L acetic, 0.62 g/L butyric and 0.64 g/L lactic acid	–	Guo et al. (2011)
Corn stover	Hydrothermal	Lignin removal of 8%	500 mL, 35 °C	Batch	1.4 g/g volatile solids (VS), 3.5 times higher	20% ethanol, 66% acetic, 8% iso-butyric acid	–	Song et al. (2019b)
Corn stover	Wet-exploded	–	3 L, 37 °C, 150 rpm, pH 6.5	Batch	1.14 g/g volatile solids (VS)	50–70% acetate, 20–45% propionate	–	Murali et al. (2017)
Rice straw	1% NaOH	–	6.5 L, pH 6, HRT 8, 9 days	Semi-continuous	1 g/g volatile solids (VS)	40–60% acetate, 20–33% propionate, 12% butyrate	Predominant class: *Clostridia*, *Bacteroidia*, *Bacteroidetes_vadin HA17* and *Deltaproteobacteria*	Lu et al. (2018)
Wheat straw	7.4% Ca(OH)_2_	Release 0.16 g Hemicellulose/g volatile solids (VS)	600 mL, 35 °C	Batch	223 mg/g volatile solids (VS)	60 mg/g volatile solids (VS) acetic, 150 mg/g volatile solids (VS) butyric acid	–	Reilly et al. (2014)
Wheat straw	–	–	6 L, lead bed reactor, 37 °C, solid-state	Batch	560 mg/g volatile solids (VS)	–	–	Rouches et al. (2019)
Palm fruit bunch	Cutting	–	40 L leach bed reactor, solid-state	Semi-continuous	196.5 mg/gTS	–	–	Saritpongteeraka et al. (2015)
Xylose	Acidic and thermal pretreatments	–	500 mL, pH 6.5, 30 °C	Batch	124 g/g volatile solids (VS), increase 187%	Acetate higher than 85%	Dominant: *Clostridia*, related with acidogenic and solventogenic processes	Mockaitis et al. (2020)

### pH

pH has been recognized as one of the most important factors for VFA production as it affects the competition between acidogens and methanogens. The optimal pH for methanogenesis is in the range of 6.6–7.5, whereas acidogens are more adaptable to a pH between 3 and 12 (Latif et al. 2017). Thus, methanogenesis can be interrupted by pH control (Cabrera et al. 2019; Jankowska et al. 2017). pH shift from neutral to less than 6.5 is a common approach to avoid methane generation and obtain higher VFA yields due to the fact that the VFA accumulation during fermentation would spontaneously reduce the pH and inhibit methane formation without extraneous supplementation of methanogenesis inhibitors. It has also been reported that overload VFA accumulation can lead to self-inhibition. Many previous studies showed that an optimal VFA production could be obtained under initial acidic or neutral conditions (Jankowska et al. 2015; Zhang et al. 2005). Zhang et al. (2005) investigated kitchen waste hydrolysis and acidogenesis under pH of 5, 7, 9 and 11. They found that pH 7 led to the highest solubilization percentage of carbohydrate, protein and lipid as well as the highest VFA yield. pH 5.5 was considered optimal for waste sludge to generate VFA at an acidic environment (i.e., pH below 7) (Latif et al. 2017). Due to the VFA consumption for methane under neutral pH (6–8) (Jankowska et al. 2015), in batch operation, methanogenesis inhibitors were usually used, while in continuous reactors, VFA accumulation was usually achieved at least after the pH has decreased from neutral value to less than 6.5 (Latif et al. 2017; Xu et al. 2021).

Given that the survival pH range for acidogens is wider than that of methanogens, an increase in pH to above 7.5 is another option for VFA production and prevention of the consumption of the produced VFA for methane formation (Jankowska et al. 2017). Cabrera et al. (2019) reported that fermentation of olive mill waste at pH 9 was 30% more effective than that at pH 5. Similarly, Wang et al. (2017) found that the efficiencies of disintegration, acidogenesis, and acetogenesis processes during sludge fermentation increased by 53%, 1030%, and 30%, respectively, by increasing the pH from 7 to 10. With regard to the contradicting views that both acidic and alkaline conditions could favor the acidogenic fermentation, another significant result reported by Jankowska et al. (2017) showed that only maize silage manifested optimal VFA production at an alkaline condition when they used maize silage, microalgae biomass and whey to produce VFA at pH 5, 7 and 11, respectively. These results sufficiently demonstrated that alkaline fermentation could enhance VFA yields from lignocellulosic biomass exclusively.

pH has also been confirmed to affect the distribution of VFA during acidogenic fermentation (Lee et al. 2014); however, no clear distribution rule has been observed due to the non-homogeneous characteristics of the different feedstocks. Generally, acetic acid, propionic acid and butyric acid account for more than 90% percentage of the total VFA compositions. Alkaline pH was found to favor the acetic-type fermentation. Chen et al. (2012) reported the proportion of acetic acid in the range of 50–75% at pH from 6 to 9 when using wetland plants as feedstocks, however, this percentage reached higher than 90% at strong alkaline conditions (pH 10–12). Zhang et al. (2012) reported that the total VFA yield from cornstalks increased by 23% and 130% when adjusted pH from 7 to 8 and 9 correspondingly, however, the enhancement only concentrated on the acetic acid yield. Similar results were also reported by Cabrera et al. (2019) that the VFA production at pH 9 was twofold higher than that of pH 5, while the increment only concentrated on acetic acid. Under neutral and acidic conditions, the production of propionic acid and butyric acid could be enhanced at individual optimal pH as well as acetic acid. Xu et al. (2021) investigated the VFA yields from corn stover at acidic pH from 5 to 8, and found a decrease of acetic acid proportion but an increase of propionic acid, although the acetate production still dominated and accounted for higher than 80% of the total VFA. Ai et al. (2014) found that a pH range of 6–6.5 contributed for butyric acid production from rice straw, with about threefold than that of pH 5–6. Jankowska et al. (2015) investigated the effect of low pH and found no significant composition variations from pH 4 to 5, regardless of a 30% increase of VFA yield. These inconsistent results could be attributed to the use of different feedstocks that required different optimal pH values for VFA production and distribution. Currently, more research is needed to summarize a VFA distribution discipline using lignocellulosic biomass as substrates. In addition, elongating the chains of VFA could achieve increase in the value-add of the VFA (Wu et al. 2019). Therefore, it is essential to explore VFA component rules under different pH and corresponding optimal conditions for VFA production. Thus, it is significant to determine the targeted acids and compositions under different pH values.

### Temperature

Temperature is an important parameter during acidogenic fermentation due to its direct involvement in microbial growth and enzyme activities. Generally, anaerobic microorganisms can function at three different temperature ranges, including psychrophilic (optimally below 20 °C), mesophilic (optimally at 35–40 °C), and thermophilic (optimally at 50–65 °C). Wang et al. (2019b) investigated the effect of temperature on VFA production from wetland plants using 5 °C as a space from 10 to 55 °C in batch operation and found that mesophilic condition was optimal for VFA generation although the production under thermophilic conditions could also be achieved by extending the fermentation period. Cavinato et al. (2017) compared the acidogenic co-fermentation from cow manure and maize silage at mesophilic and thermophilic conditions, and found fermentation at 37 °C range leading to 30% higher VFA production while about 50% higher solubilization yield at 55 °C range. However, due to this enhanced hydrolysis ability at higher temperature, thermophilic condition was found in favor of the fermentation with solid-state or high solid concentration, i.e., 30 g volatile solids (VS)/L (Achinas and Euverink 2020; Shi et al. 2013).

### Organic loading rate and retention time

Organic loading rate (OLR) refers to the amount of substrate fed into the reactor per day per working volume. A moderate increase of OLR definitely improves VFA productivity, and an excessively high OLR tends to lead to VFA accumulation, inhibiting both the methanogenic and acidogenic microorganisms. Jiang et al. (2013) investigated the effects of three different OLR, namely, 5, 11, and 16 gTS/(L·d). They found that VFA yields increased from 11 g/L to 17 and 22 g/L, respectively, during the first week at each OLR. However, while VFA production continued for OLR of 11 gTS/(L·d), that for OLR of 16 gTS/(L·d) slumped from day 10. Upon decreasing the OLR from 16 to 10 gTS/(L·d), the reactor returned to a stable VFA production of 14 g/L. A common OLR range of 1.2–12 g volatile solids (VS)/(L·d) for AD could also provide some guidance to perform the acidogenic fermentation (Li et al. 2015). The optimal OLR, however, might vary with the substrates, temperature, and hydraulic retention time. For instance, a higher OLR could yield more VFA at a higher temperature (Gou et al. 2014). Lignocellulose acidogenesis tended to burden a higher OLR than food waste or sludge due to the lower viscosity of the fermentation broth (Wainaina et al. 2019a).

Hydraulic retention time (HRT) is the duration that the feedstock is kept within the reactor. It is an important parameter for continuous reactor and pilot-scale operations. In acidogenic fermentation, VFA production could dramatically increase during the first 10 days and then reach a steady-state production state (Jiang et al. 2013). On the other hand, an HRT of approximately 8–20 days was required for methanogenesis (Wikandari and Taherzadeh 2019). Thus, 8–12 days could be an appropriate HRT for considerable VFA production and avoiding its bioconversion into methane. While there is no doubt that a longer HRT could lead to more soluble organic matters, Wainaina et al. (2019a) reported that an increase of HRT to 20–30 days did not further improve VFA production, even though the higher HRT slightly increased the percentage of butyrate and acetate.

### Methanogenesis inhibitors

Another option to prevent VFA consumption for methanogenesis is to deactivate the methanogens using chemical inhibitors and coenzyme M analogs, including 2-bromoethanesulfonate (BES), 2-mercaptoethanesulfonate (MES), and lumazine. Coenzyme M is involved in the terminal step of methane synthesis, where the methyl group carried by coenzyme M is reduced to methane by reductase (Liu et al. 2011). Inhibitor analogs competitively function to inhibit the methyl transfer and interrupt the methane-forming process from CO_2_/H_2_ and acetate. Typically, a varying concentration of 10–50 mg/L BES has been reported to inhibit methanogenesis (Liu et al. 2011). Although it seems that pH control could also work in tandem with the addition of inhibitors to inhibit the formation of methane, typically inhibitors were used in reactors without pH control (initial neutral pH) (Jankowska et al. 2017). Eryildiz and Lukitawesa (2020) observed that under an acidic condition with a pH of 4–6, the addition of chemical inhibitors did not make a significant change in the amount of VFA produced. On the other hand, it is important to recognize that the usage of methanogenesis inhibitors would inevitably increase the operating cost of the process. For example, if we adopt Jankowska’s experimental methods as reference (Jankowska et al. 2017), the cost using BES inhibitors is about 0.252 USD/g volatile solids (VS) with 0.44 gVFA/gCOD yield, and the cost using alkaline fermentation is about 0.076 USD/g volatile solids (VS) with 0.70 gVFA/gCOD yield. Other cost-effective methanogenesis inhibitions like pH control and wash-out of methanogens are discussed in “Alkaline fermentation” and “High OLR but short HRT” sections, respectively.

## Proposed strategies for VFA production

### Optimization of operating parameters

#### Alkaline fermentation

To maximize VFA production, it is necessary to enhance VFA accumulation and simultaneously prevent its consumption by judiciously controlling the pH. As previously mentioned in “pH” section, there have been some disputes in the optimal pH for acidogenic fermentation. Specifically, both acidic and alkaline conditions were found effective to improve the VFA yield during fermentation of sludge, waste water, and food waste. Jankowska et al. (2017) used maize silage, microalgae biomass and whey to produce VFA at pH 5, 7 and 11, respectively, and found that only maize silage manifested optimal VFA production at an alkaline condition (pH 11). Similar enhanced results were observed from fermentation of olive mill waste at pH 9 compared to pH 5 (Cabrera et al. 2019). Park et al. (2014) demonstrated that the alkaline pH improved the hydrolysis of organic matter and further provided soluble substrates for the acidogenic microorganisms for the production of the VFA. Based on these findings, it seems that alkaline conditions favor VFA production especially for lignocellulosic biomass wastes because of its composition and recalcitrant structure against hydrolysis. Thus, due to the different characteristics among varieties of feedstocks, it is essential that pH control be individually evaluated to facilitate acetogenesis. Although the initial pH strongly affected the VFA production, the starting alkaline pH did not sustain and always tended to drop to neutral level unless the pH had been controlled (Jankowska et al. 2017). For semi-continuous or pilot-scale demonstrations, NaOH and Ca(OH)_2_ were usually used to adjust the pH to maintain alkaline conditions during fermentation (Gao et al. 2011; Li et al. 2011). Several pilot-scale and full-scale applications using sludge as feedstocks can be found (Da Ros et al. 2020; Li et al. 2011; Liu et al. 2018). Currently, since most studies focused on batch-mode fermentation rather than semi-continuous or continuous fermentation, the effect of extra alkali on the microbes remains unclear. Usage of alkali to adjust reactor pH would concomitantly increase operating cost. To minimize operating cost, a combination of alkaline fermentation and alkaline pretreatment might be an option.

### Co-digestion and solid-state fermentation

C and N elements are important nutrition for the metabolism of microbes. A high C/N ratio of lignocellulosic biomass wastes could not supply adequate N nutrition for microorganisms, which limits the fermentation rate and even causes insufficient consumption of the C source. An appropriate C/N ratio is essential to maintain the nutrition balance within the reactor. A C/N ratio of 25 has been considered optimal for anaerobic fermentation (Wang et al. 2012). In this case, co-digestion of lignocellulosic biomass wastes with others biomass wastes with low C/N ratios, like animal manure, food waste, and sludge (Mu et al. 2020; Wang et al. 2018; Zhang et al. 2018), to adjust the C/N ratio could significantly enhance microbial activities and VFA production. Macias-Corral et al. (2008) investigated co-digestion of agricultural waste and cattle manure, and obtained around 20% higher soluble COD increased from 27,000 to 34,000 mg/L, 60% higher VFA yield increased from 8 to 13 g/L. Zhang et al. (2018) added equivalent food waste into yard waste for co-digestion and also obtained an enhanced SCOD production from 16,000 to 35,000 mg/L in the first week. Indeed, food waste augmentation is a common approach in co-digestion. It had been reported that lignocellulose tended to release carbon sources slowly and limited the start-up phase, while food waste rapidly got acidified and provided available carbon for fermentation (Wang et al. 2018). On the other hand, mono-digestion of food waste could result in the decrease in pH to around 3.5 (Zhang et al. 2017). The acids generated during hydrolysis facilitated acid hydrolysis pretreatment of the lignocellulosic biomass wastes within the fermenter. Furthermore, co-digestion is also popular in practical biogas or fermentation plant due to its low cost and simple operation (André et al. 2019; Lansing et al. 2019).

Initial feedstock concentration has also been regarded as a factor to affect fermentation performance. In conventional AD, 6–8% TS is usually regarded as the optimal initial substrate concentration for methane production (Holliger et al. 2016). To improve organic loading and shorten the fermentation period, solid-state AD with a TS above 15% had been suggested. However, solid-state AD had revealed several disadvantages such as low methane yields and relatively high process instability due to the fact that high TS contents could limit the methanogenesis and led to VFA accumulation (Yang et al. 2015). In such case, solid-state AD might be expected to be an enhancing strategy to convert lignocellulose into VFA rather than methane. Rouches et al. (2019) investigated the impact of substrate/inoculum ratio of 1.2, 2.0, 3.6 and 8.5, and the ratio of 8.5 group obtained highest yield of 10 g/L while the VFAs of other groups declined for methane formation. In another study, Fang et al. (2018) also reported successfully using lignocellulosic solid digestate for VFA production in solid-state fermentation (TS of 15%), and obtained the VFA yield of 250 mgCOD/g volatile solids (VS). Taken together, high solid-state fermentation seems a feasible way to convert lignocellulosic biomass wastes into VFA. A concerned problem associated with solid-state fermentation might be VFA extraction and application. In fact, TS in solid-state fermenter were usually 15%, thus, the broth is close to fluid. Rouches et al. (2019) designed a leachate tank at the bottom in the solid-state fermenter to collect VFA, which could successfully work at solid/inoculum ratio from 1.2 to 8.5. Similar pilot-scale reactor could also be seen in other publications (Puyuelo et al. 2010; Saritpongteeraka et al. 2015). These reports sufficiently indicated that a liquid-phase reservoir at the base of reactors could collect the VFA and even recycle the leachate. However, the results of solid-state acidogenic fermentation remain inadequate. Meanwhile, the enhancement of reactor stability during long-term fermentation also needs to be investigated in the future.

#### High OLR but short HRT

Given that pH during fermentation would inherently drop and increased OLR would aggravate the acidification (Jankowska et al. 2017), proper OLR control could reduce pH to less than 6.5 and successfully enhance the VFA yields. Besides, growth of methanogens is slower than acidogens. Hence, a shorter HRT could also achieve the wash-out of methanogens, avoid consumption of VFA and improve the treatment capacity. This approach was used to investigated co-fermentation of maize silage and cow manure, and found that the VFA yield reached 150, 183 and 162 gCOD/kg volatile solids (VS) at HRT of 2, 4 and 6 days, respectively (Cavinato et al. 2017). The same article also indicated the lower soluble COD, which could be interpreted as the consumption for methane. Similarly, an HRT of 10 days was also adopted to investigate the acidogenic fermentation of sugarcane filter cake in semi-continuous operation, and the VFA yield could reach 320 mg/g volatile solids (VS) in steady stage. A shorter HRT of 1.5 days was used to investigate the effect of pH during wash-out operation (Cysneiros et al. 2012). The results showed that the VFA yield of experimental group without pH buffer was lower than controlled group (no wash-out), which could be explained by a possible wash-out of acidogens in the leachate and the growth rate of acidogens did not compensate for the wash-out rate; but, the experimental group with pH buffer (pH 6.5) enhanced VS degradation and VFA yield by 15% and 32%, respectively. Additionally, the process of inoculum washing-out incredibly led to the decrease of NH_4_-N, which could serve to buffer the reactor’s pH and balance the low nitrogen solubilization from lignocellulosic substrates (Janke et al. 2016). After nitrogen supplementation, the pH value increased from 5.2 to 6.7, but the VFA production decreased to the lowest level due to the consumption of methanogenesis at HRT of 5 days (Janke et al. 2016). Moreover, a high OLR and a short HRT frequently cause insufficient degradation of lignocellulose and a low mass transfer rate. Thus, an appropriate OLR and HRT should be experimentally determined associated with pH and various lignocellulosic feedstocks.

### Combination of effective pretreatments

Feedstock pretreatment is one of the most effective ways to enhance fermentation performance, especially for lignocellulosic biomass wastes due to their obstinate structures for hydrolysis. But the single cost of pretreatments is approximately as high as other production processes (Soltanian et al. 2020). Thus, it is essential to seek cost-effective pretreatments to balance pretreatment cost and VFA production. Typically, mono-pretreatment is not the most effective and economic method in AD. Instead, a combination of two or multiple pretreatment methods to pretreat the feedstocks is usually adopted. As VFA are intermediate compounds during AD, it is reasonable that the pretreatment methods on AD can be applied for acidogenic fermentation. Previously, we had summarized the pretreatments on organic wastes for biogas production via AD (Zhang et al. 2019). In this review, we intend to highlight some novel and cost-effective methods with a focus on co-pretreatments. Pretreatments are usually classified into four categories, including physical, chemical, biological pretreatments, and co-pretreatments (Table 3). Concerning the characteristics of lignocellulosic biomass wastes, size reduction is usually necessary before several chemical or biological pretreatments. After biomass harvest or collection, the size of the lignocellulosic biomass is typically large and consequently present unfavorable area-to-mass ratio for hydrolysis. Previous studies showed that researchers are accustomed to mill the biomass into less than 2 mm or even smaller, which may not be economical and practical for large-scale feedstocks. To mill or cut lignocellulosic biomass into 1 cm size usually could be achieved in biogas plants, but the energy needed for further particle size decrease would be multiple folds and not cost-effective (Vidal et al. 2011). For example, energy of 28–35 and 14 kWh/t were required to mill corn stover with hammer to 3.2 and 1.6 mm, respectively, however, the digestibility had no difference with dilute alkali pretreatment when the particle size was less than 2 mm (Vidal et al. 2011). Similarly, Sun et al. (2019) used liquid digestate to pretreat wheat husk with different particle sizes of 2 mm, 5 mm and 8 mm and found this negligible effect of particle reduction when increased pretreatment time from 3 to 5 days. The maximum biomass sizes which no further increase in pretreatment effectiveness might vary with the co-pretreatment approaches (Vidal et al. 2011). In addition to milling, shearing and cutting are good alternative to size reduction since the structures of lignocellulose will not be damaged. Thus, acceptable pretreatments, parameter optimization or co-pretreatments could off-set the cost-intensive particle size reduction process for treating large amounts of feedstocks. Among physical pretreatment technologies, the use of microwave energy, which relies on an applied electromagnetic field to generate heat is popular. Microwave heating was more rapid and less energy intensive compared to convection heating and could favorable enhance the lignocellulosic structure amenable to hydrolysis (Hu and Wen 2008).

It has been known that alkali addition [e.g., NaOH and Ca(OH)_2_] is the most effective chemical pretreatment and it has been confirmed in industrial applications, regardless of their known disadvantages of land pollution. Thus, co-pretreatment of microwave and alkali addition has also been investigated, even though the augmentation with microwave did not exhibit the synergistic effect anticipated with co-alkaline pretreatment compared to mono-alkaline pretreatment. More specifically, co-pretreatment could effectively accelerate the hydrolysis process, but could not further enhance VFA production (Elalami et al. 2020). Hydrothermal pretreatment, previously operated at 170–230 °C (Saha et al. 2013) and negatively regarded due to its high energy input demand, has recently attracted more interest due to the fact that the enhancement of hydrothermal pretreatment at a lower temperature has been observed. Xiang et al. (2021) used hydrothermal pretreatment to pretreat rice straw at 90–130 °C for 15 min and enhanced the VFA yield by 38%. Even a low temperature (i.e., 50 °C) pretreatment with a prolong pretreatment time of 24 h led to a 31% of enhancement (Yuan et al. 2019). Due to its advantages of simple operation, involving only water, and no waste generation, hydrothermal pretreatment is gaining more traction. The use of H_2_O_2_ was also adopted for pretreatment on varieties lignocellulosic biomass wastes, like wheat straw, corn stover, and rice straw (Gould 1984; Kocher et al. 2017; Liu et al. 2019a). It was touted as a potential pretreatment method because of the generation of zero residues from the degradation of H_2_O_2_. The use of H_2_O_2_ pretreatment has also been combined with microwave and alkaline pretreatment methods. During the alkaline H_2_O_2_ pretreatment, H_2_O_2_ played the role of an oxidant and the alkali functioned to reduce or remove 30–40% lignin and acetyl in the hemicellulose, so that the accessibility and digestibility of the hemicellulose was enhanced by 30% (Sun et al. 2015). On the other hand, the lignin-oxidizing species was a highly reactive hydroxyl radical (HO^·^), formed during the degradation of H_2_O_2_ in the reaction with the hydroperoxyl anion (HCOO−), where hydroperoxyl and hydroxyl radicals were both responsible for solubilizing hemicelluloses (Perendeci et al. 2018). Microwave pretreatment with H_2_O_2_ could increase decomposition of H_2_O_2_ into HO^·^ radical under more acceptable heating temperature (< 100 °C), therefore enhancing the oxidation of H_2_O_2_ (Liu et al. 2016b). The results showed the synergetic pretreatments at 100 °C released the double soluble sugars, and the synergetic effect at 80 °C was equivalent to that of mono-microwave pretreatment at 120 °C (Eskicioglu et al. 2008). Similarly, H_2_O_2_ had been reported to combine with UV, leading to a delignification rate of 76.6% and reducing the H_2_O_2_ charge of 0.4 g/g substrates, compared with alkaline H_2_O_2_ pretreatment when using sisal waste as feedstocks (Yang et al. 2018).

Among biological pretreatments, liquid digestate has been investigated for enhancement of lignocellulosic biomass wastes for biofuel production (Sun et al. 2019; Zhang et al. 2018). Liquid digestate, which refers to sludge discharged from digesters, is considered a biological pretreatment medium due to that it contains both anaerobic microbes and ammonia that can specifically degrade or acidify the substrates. Liu et al. (2019c) found a decrease of cellulose and hemicellulose contents from 39 to 26% and from 31 to 24%, respectively, when using liquid digestate to pretreat wheat straw for 5 days. They had argued that it was ammonia in the liquid digestate that played a major role in such a pretreatment process. Indeed, they used liquid digestate and ammonia solution with the same ammonia concentration to pretreat wheat straw and obtained similar enhanced yields.

At present, lignocellulosic biomass valorization, which involved the conversion of cellulose and hemicellulose into biofuels or chemical materials, lignin valorization has largely been unexplored. For example, lignin could be further used as composites, plastics, and chemicals (Ragauskas et al. 2014). It might be possible to separate hemicellulose and cellulose for acidogenic fermentation while lignin separated be used for other high value-added conversions. Available approaches in this regard are divided into two ways, namely, hydrolysis of polysaccharides and subsequent recovery of lignin or initial solubilization of lignin, leaving sugars as the solid residue for acidogenic fermentation (Azadi et al. 2013; Garedew et al. 2020). Song et al. (2019b) used alkaline-oxygen pretreatment to solubilize lignin as adsorbents while cellulose and hemicellulose were then hydrolyzed for fermentation sugars. Dilute acid hydrolysis could solubilize the polysaccharides to their monomeric constituents while the lignin would exist in the solid fraction to achieve the fractionation (Azadi et al. 2013). Lignin was also reported to be separated from lignocellulosic biomass wastes at 200 °C with hydrothermal pretreatment and hydrogen catalyst (Azadi et al. 2012). Overall, appropriate pretreatments should be selected on account of their operation costs, enhancement efficiencies, and desired final products (Table 3).

**Table 3:** Pretreatment of lignocellulosic biomass

Pretreatment	Substrates	Conditions	Conclusion	Advantages and suggestion	References
Mill and liquid digestate soaking	Wheat husk	2–8 mm, soaked for 1–5 days	The yields were similar of different size-reduction for 5-day soaking	Co-pretreatment to off-set the cost for particle size reduction	Sun et al. (2019)
Microwave-alkali	Olive	200–700 W for 2 min, 4% NaOH for 4 h	Co-pretreatment shortened the hydrolysis, but not further enhanced VFA yield	Feasible for short HRT fermentation	Elalami et al. (2020)
Hydrothermal	Corn stover	170–230 °C for 5–60 min	Highly rely on temperature than duration time	Low temperature hydrothermal was feasible, but longer duration needed	Saha et al. (2013)
Hydrothermal	Rice straw	90–130 °C for 15 min	VFA yield improved by 38%; low temperature hydrothermal was effective		Xiang et al. (2021)
Hydrothermal	Corn stover	50 °C for 24 h	31% yield enhancement; low temperature hydrothermal was effective		Yuan et al. (2019)
Alkaline H_2_O_2_	Crop stalks	1–5% H_2_O_2_ at pH 11.5	Remove 30–40% lignin and increase 8% digested fraction	Clean pretreatment and Zero residues	Sun et al. (2015)
Microwave- H_2_O_2_	Forest waste	2.5 M H_2_O_2_ at 400 W	Increase 12% cellulose release compared to mono- H_2_O_2_ pretreatment		Camani et al. (2020)
UV-H_2_O_2_	Sisal waste	0.1 g H_2_O_2_/g Substrate, UV for 6 h	Led to delignification rate of 76.6%, increasing by 25% than mono-H_2_O_2_ pretreatment		Yang et al. (2018)
Liquid digestate soaking	Wheat husk	Soaked for 5 days, open air	Increased 30% yield	Cost-saving, digestate recycle	Sun et al. (2019)
Liquid digestate soaking	Wheat straw	Soaked for 5 days, open air	Ammonia in digestate functioned		Liu et al. (2019a)
Lignocellulose fractionization	Wood pellets	18% (w/w) alkali, 100psi O_2_ for 6 h	Remove 52% lignin	Lignocellulose fractionization for valorization	Song et al. (2019a, b)
Deep eutectic solvents	Wood	120 °C for 2 h	Remove high to 78% lignin		Alvarez-Vasco et al. (2016)
Deep eutectic solvents	Wheat straw	110 °C for 2 h	Hydrolyzed 90% cellulose and 71% xylan		Liu et al. (2019b)
Deep eutectic solvents	Wood shavings	130 °C for 3 h	Remove 79% lignin		Tian et al. (2020)

### Reactor upgrading

#### Coupled electrochemical technologies with AD

Extracellular oxidation reduction potential (ORP) is an important parameter because it could influence intracellular ORP and NAD^+^/NADH ratio in microbial cells, affecting gene expression and enzyme synthesis for overall metabolic activity. In this regard, electrical current supply can regulate ORP and the NAD^+^/NADH ratio to further enhance microbial activities. An electrochemical system usually consists of an anode and cathode that are separated by an ion selective membrane to form two individual chambers (De Vrieze et al. 2018). Electrochemical fermentation is microbe-derived rather than current-derived, thereby requiring a relatively low current density (0.001–10 A/m^2^) (Sturm-Richter et al. 2015). For example, it has been reported that 99.8% electrons originated from the substrate while only 0.2% electrons were supplied from external power (Choi et al. 2015). Furthermore, chain elongation of fatty acid via electrochemical fermentation with increased value of VFA had also been observed (Jiang et al. 2020).

**Fig. 4:**
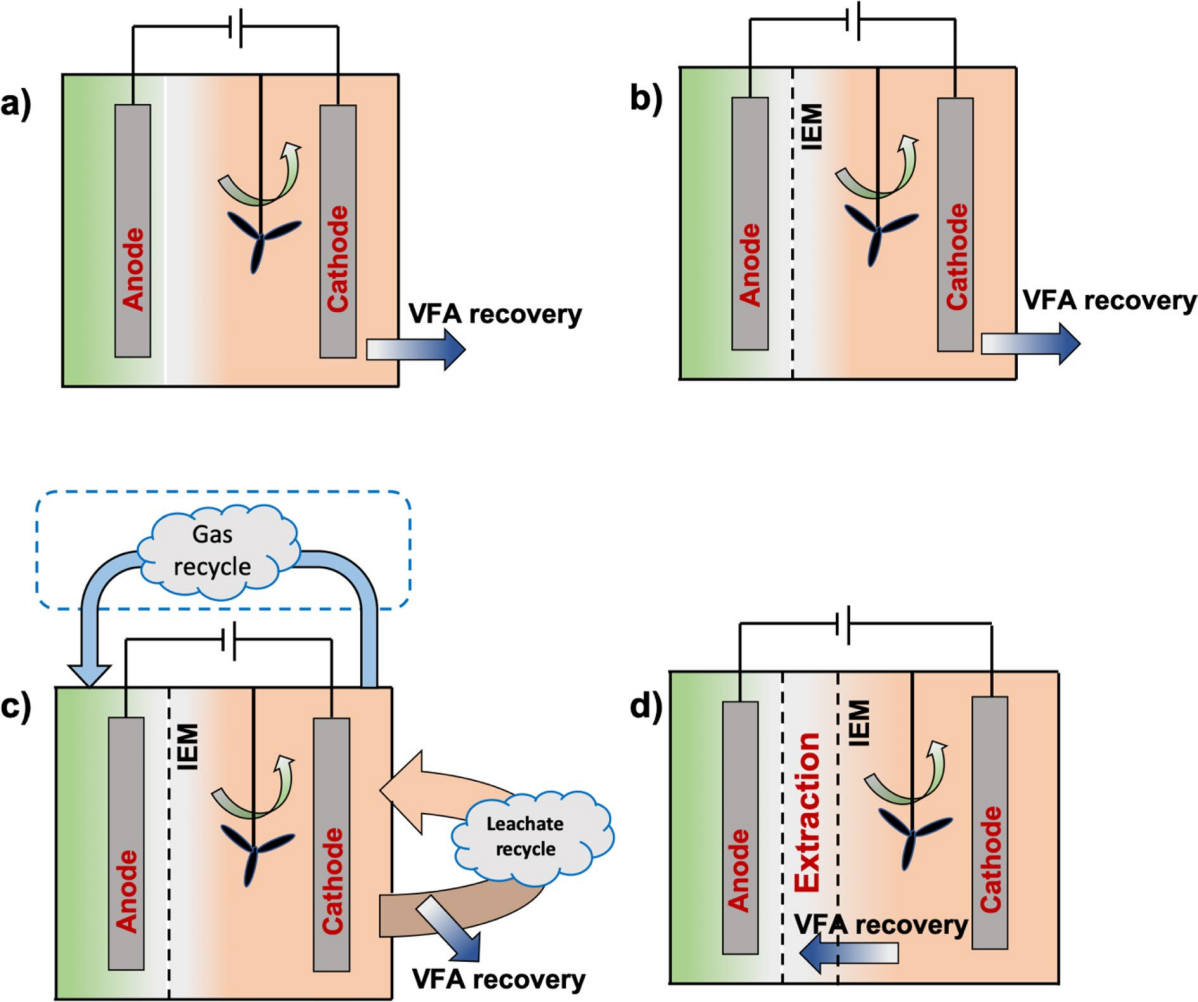
Possible electrochemical fermentation reactors: **a** single chamber without membrane separation; **b** membrane-separated chambers; **c** reactor with off-gas recycle, and **d** reactor with membrane extraction chamber (IEM: ion exchange membrane)

Ion exchange membrane (IEM) was used to separate the reactor into two or even several chambers, while lignocellulosic biomass wastes as the substrate was placed both in the cathode chamber for reduction and the anode chamber for oxidation (Jiang et al. 2019) (Fig. 4). VFA or off-gas recycle system could be coupled to the reactor to enhance VFA production and extraction. Andersen et al. (2014) designed a recycle system where VFA could flow through an anion exchange membrane from a cathode chamber into an anode chamber, and then the aqueous carboxylic acid concentrate reacted with added alcohol in a water-excluding phase to generate volatile esters in order to achieve VFA extraction. Zhou et al. (2019) recycled by-product CO_2_ and H_2_ generated in an anode chamber to a cathode chamber where the off-gas could further form acetic acid. This arrangement increased VFA production and alleviated inhibition due to excess H_2_ pressure. Furthermore, the use of two IEM in a reactor can form another extraction chamber to achieve in situ VFA extraction and avoid VFA accumulation. In general, the working- and counter-electrodes and their respective reactions can take place in either single or separated chambers. If the counter-reaction products are compatible with the purity of the working-reaction products, the single-chamber reactor is ideal, and the simple incorporation of traditional fermenters with electrodes can act as electrochemical fermentation systems (Rago et al. 2019). Commonly used materials for the electrodes are carbon and graphene materials due to their high conductivity, good chemical stability, and relatively low costs. To improve electron transfer efficiency, carbon rod had been substituted with carbon felts or brush to facilitate biofilm formation (Guo et al. 2017; Zhou et al. 2019). The use of horizontally oriented electrodes in upflow anaerobic bioreactors with built-in electrochemical system had also been investigated. It has been found that the optimal design in such a case occurred when the working electrode was placed at the bottom (Gao et al. 2020). However, most of current researchers only focused on lab-scale reactors (e.g., volume less than 500 mL) (Jiang et al. 2019) and only wastewater, glucose or food waste have been utilized as substrates to perform electrochemical fermentation (Jiang et al. 2020; Liu et al. 2019b; Zhou et al. 2019). The effectiveness of the AD–electrochemical integrated system fed with lignocellulosic biomass wastes remains unclear and needs to be investigated. Moreover, performance of large-scale reactors should also be investigated operated under continuous mode.

#### VFA recovery

The shortage of industrial VFA-based processes is partly attributed to the difficulty in recovering the VFA from digestate and low product yields. All of the literature reviewed above had focused on optimizing the operating conditions with regards to VFA production. It is also well-known that high VFA accumulation could also inhibit the activity of acid-producing bacteria. Membrane technology has been the most researched VFA recovery techniques. The membrane setup can be placed either within or outside of the bioreactors (Musa et al. 2018). Based on a driving force characterization, the membrane bioreactor setup can be classified as pressure-driven, such as the use of microfiltration and nanofiltration membranes, electrically driven, such as electrodialysis, or vapor pressure difference/concentration gradient driven. Currently, the inbuilt gas diffuser reactors were widely researched for membrane cleaning (Wainaina et al. 2019b). Due to the lower viscosity of lignocellulosic particles compared with sludge, membrane fouling might be further alleviated. Wainaina et al. (2019b) designed an immersed membrane reactor incorporating 12 inbuilt gas diffusers to achieve in situ VFA recovery and robust cleaning capacities. Khan et al. (2019) also investigated pH effect on acidogenic fermentation in a membrane reactor and found that acid and alkaline conditions tended to cause membrane fouling. Liu et al. (2016a) invented a self-form dynamic membrane formed by the precipitation of microorganisms and their metabolites during the filtration of activated sludge, with advantages of low filtration pressure and cheap membrane materials. Aydin et al. (2018) used vapor permeation membrane contactors to drive unionized VFA flow from feed solution into permeate solution, where alkaline conditions could dissociate VFA into their ionic forms, thereby creating zero pressure in in the permeate side of the membrane. In addition, liquid membranes based on lipophilic amines and biodiesel have also been proposed because some organic extractants could selectively separate VFA (Torri et al. 2019). In the long run, membrane technology has the potential for continuous anaerobic fermentation. Reduction of membrane costs and fouling are two major operating issues that need to be solved.

Appropriate VFA recovery technologies are associated with the fermentation operation, product compositions and final target. Free acid form (pH < pKa) favors membrane recovery, so that longer chain acids, i.e., butyric acid trend to be sustain and recovered (Ramos-Suarez et al. 2021). In terms of specific alkaline fermentation of lignocellulosic feedstocks, electrodialysis technology was made available for recovering the acids in the dissociated form (pH > pKa). Electrodialysis technology also demonstrated a preference towards acetic acid recovery compared to other VFAs (Pan et al. 2018). This would be advantageous for VFA recovery from alkaline fermentation, for that acetic acid dominated the VFA compositions in alkaline pH.

## Techno-economics and industrial perspectives

In anaerobic fermentation, there are more diverse products derived from VFA than biogas, opening new markets for value-added chemicals and achieving lignocellulose valorization. Regardless of the derivatives, the prices of acetic acid, propionic acid and butyric acid are in the range of (USD/tonne) 500–850, 1500–2300 and 1800–1900 USD/t, respectively, while the price of methane is only about 150–600 USD/tonne (Baumann and Westermann 2016). One concerned problem is the cost associated with extraction and purification of the mixed VFA broths. The maximum purification cost might be about 15 USD/m^3^ (Ramos-Suarez et al. 2021). One economic assessment for food waste fermentation had indicated that acidogenic fermentation could potentially produce more revenues than biogas upgrading, and the highest profit could be as high as 296 USD/tVS compared to only 19 USD/tVS for anaerobic digestion (Bastidas-Oyanedel and Schmidt 2018). No analysis and results about lignocellulosic acidogenic fermentation are found in the literature to date. The economics will also vary depending on the specific process operation, acidogenic efficiency and the market.

The minimum selling price of the mixed VFAs from brown algae was calculated at about 384 USD/t, which was lower than the market price for (petrochemical) acetic acid (Fasahati and Liu 2014). VFA downstream purification is therefore quite essential for profitability. At present, the most researched VFA applications are not limited to bioplastics, bio-oil (biodiesel), chain elongation, electric power and biological nutrients removal (Lee et al. 2014). To date, the reported full-scale VFA applications only focus on biological nutrients removal with sludge or waste water as feedstocks (net profit of 9.12 USD/t) (Liu et al. 2018; Andalib et al. 2017). The possible reason might be attributed to the present uncompetitive revenues. The techno-economic analysis of bioplastic (PHB) production from VFA showed that the routine is not yet competitive with those for the petrochemical counterparts (Fernández-Dacosta et al. 2015). The results on lignocellulose fermentation are rare. But still, the wish of green economy encourages us to explore and optimize our operating processes.

## Conclusions and recommendations for future work

In this review, we have identified the characteristics of lignocellulosic biomass wastes and their typical metabolism pathways of acidogenic fermentation. Following that, the effects of several key operating conditions were discussed and some available parameter optimization approaches were presented. In addition, parameter optimization and effective enhancing strategies have also been discussed, including pretreatments, electrochemical fermentation, and membrane bioreactors. Main conclusions and the related recommendations for future work included:

1. A workflow chart for parameter optimization and enhancing strategies to guide the operation of acidogenic fermentation. Appropriate parameter regulation requires acceptable particle size, mesophilic temperature (35–40 °C), C/N ratio (20–40), OLR (< 12 g volatile solids (VS)/(L·d)), and the maximum HRT (8–12 days);
2. Consideration of acidic, neutral and alkaline pH conditions to enhance VFA production with various feedstocks. In particular, alkaline fermentation carries high potential for lignocellulosic biomass fermentation. pH regulation through appropriate pH control and gradual increase of OLR can be a promising way to prevent VFA conversion to methane;
3. Co-pretreatments can be adopted to avoid the cost-intensive particle size reduction process. Combination with chemical pretreatments using alkali, H_2_O_2_, and digestate is beneficial for lignocellulosic biomass hydrolysis, as well as separation of cellulose and hemicellulose from the lignin structures;
4. Electrochemical fermentation is a novel technology which has been demonstrated using wastewater, glucose, and sludge as substrates. Currently, the studies of electrochemical fermentation with lignocellulosic biomass as feedstocks remain lacking, but definitely worth exploring;
5. Membrane technology plays an important role in extraction of the accumulated VFA and the use of several membrane bioreactors has been reported. In addition to membrane bioreactors, other in situ and ex situ VFA extraction technologies deserve more exploration in the future.

## Data Availability

Not applicable.

## Abbreviations

VFA: Volatile fatty acids
AD: Anaerobic digestion
OLR: Organic loading rate
HRT: Hydraulic retention time
ORP: Oxidation reduction potential

## References

[CR1] Achinas S, Euverink GJW (2020). Effect of temperature and organic load on the performance of anaerobic bioreactors treating grasses. Environments.

[CR2] Agematu H, Takahashi T, Hamano Y (2017). Continuous volatile fatty acid production from lignocellulosic biomass by a novel rumen-mimetic bioprocess. J Biosci Bioeng.

[CR3] Ai B, Li J, Chi X (2014). Effect of pH and buffer on butyric acid production and microbial community characteristics in bioconversion of rice straw with undefined mixed culture. Biotechnol Bioprocess Eng.

[CR4] Alvarez-Vasco C, Ma R, Quintero M (2016). Unique low-molecular-weight lignin with high purity extracted from wood by deep eutectic solvents (DES): a source of lignin for valorization. Green Chem.

[CR5] Andalib M, Taher E, Money B (2017). Full scale demonstration of non-VFA pathway enhanced biological phosphorus removal. Proc Water Environ Fed.

[CR6] Andersen SJ, Hennebel T, Gildemyn S (2014). Electrolytic membrane extraction enables production of fine chemicals from biorefinery sidestreams. Environ Sci Technol.

[CR7] André L, Zdanevitch I, Pineau C (2019). Dry anaerobic co-digestion of roadside grass and cattle manure at a 60 L batch pilot scale. Bioresour Technol.

[CR8] Aydin S, Yesil H, Tugtas AE (2018). Recovery of mixed volatile fatty acids from anaerobically fermented organic wastes by vapor permeation membrane contactors. Bioresour Technol.

[CR9] Azadi P, Carrasquillo-Flores R, Pagán-Torres YJ (2012). Catalytic conversion of biomass using solvents derived from lignin. Green Chem.

[CR10] Azadi P, Inderwildi OR, Farnood R, King DA (2013). Liquid fuels, hydrogen and chemicals from lignin: a critical review. Renew Sustain Energy Rev.

[CR11] Bastidas-Oyanedel J-R, Schmidt J (2018). Increasing profits in food waste biorefinery—a techno-economic analysis. Energies.

[CR12] Baumann I, Westermann P (2016). Microbial production of short chain fatty acids from lignocellulosic biomass: current processes and market. BioMed Res Int.

[CR13] Cabrera F, Serrano A, Torres Á (2019). The accumulation of volatile fatty acids and phenols through a pH-controlled fermentation of olive mill solid waste. Sci Total Environ.

[CR14] Camani PH, Anholon BF, Toder RR, Rosa DS (2020). Microwave-assisted pretreatment of eucalyptus waste to obtain cellulose fibers. Cellulose.

[CR15] Cavinato C, Da Ros C, Pavan P, Bolzonella D (2017). Influence of temperature and hydraulic retention on the production of volatile fatty acids during anaerobic fermentation of cow manure and maize silage. Bioresour Technol.

[CR16] Chen Y, Wen Y, Zhou J (2012). Effects of pH on the hydrolysis of lignocellulosic wastes and volatile fatty acids accumulation: the contribution of biotic and abiotic factors. Bioresour Technol.

[CR17] Choi O, Kim T, Woo HM, Um Y (2015). Electricity-driven metabolic shift through direct electron uptake by electroactive heterotroph *Clostridium pasteurianum*. Sci Rep.

[CR18] Corneli E, Dragoni F, Adessi A (2016). Energy conversion of biomass crops and agroindustrial residues by combined biohydrogen/biomethane system and anaerobic digestion. Bioresour Technol.

[CR19] Cysneiros D, Banks CJ, Heaven S, Karatzas K-AG (2012). The effect of pH control and ‘hydraulic flush’ on hydrolysis and volatile fatty acids (VFA) production and profile in anaerobic leach bed reactors digesting a high solids content substrate. Bioresour Technol.

[CR20] Da Ros C, Conca V, Eusebi AL (2020). Sieving of municipal wastewater and recovery of bio-based volatile fatty acids at pilot scale. Water Res.

[CR21] De Vrieze J, Arends JBA, Verbeeck K (2018). Interfacing anaerobic digestion with (bio)electrochemical systems: potentials and challenges. Water Res.

[CR22] Elalami D, Carrere H, Abdelouahdi K (2020). Mild microwaves, ultrasonic and alkaline pretreatments for improving methane production: impact on biochemical and structural properties of olive pomace. Bioresour Technol.

[CR23] Eryildiz B, Lukitawesa TMJ (2020). Effect of pH, substrate loading, oxygen, and methanogens inhibitors on volatile fatty acid (VFA) production from citrus waste by anaerobic digestion. Bioresour Technol.

[CR24] Eskicioglu C, Prorot A, Marin J (2008). Synergetic pretreatment of sewage sludge by microwave irradiation in presence of H_2_O_2_ for enhanced anaerobic digestion. Water Res.

[CR25] Fang W, Zhang P, Zhang X (2018). White rot fungi pretreatment to advance volatile fatty acid production from solid-state fermentation of solid digestate: efficiency and mechanisms. Energy.

[CR26] Fasahati P, Liu J, Eden MR, Siirola JD, Towler GP (2014). Techno-economic analysis of production and recovery of volatile fatty acids from brown algae using membrane distillation. Computer aided chemical engineering.

[CR27] Fernández-Dacosta C, Posada JA, Kleerebezem R (2015). Microbial community-based polyhydroxyalkanoates (PHAs) production from wastewater: techno-economic analysis and ex-ante environmental assessment. Bioresour Technol.

[CR28] Galbe M, Zacchi G (2012). Pretreatment: the key to efficient utilization of lignocellulosic materials. Int Conf Lignocellul Ethanol.

[CR29] Gao Y, Peng Y, Zhang J (2011). Biological sludge reduction and enhanced nutrient removal in a pilot-scale system with 2-step sludge alkaline fermentation and A2O process. Bioresour Technol.

[CR30] Gao L, Thangavel S, Guo Z-C (2020). Hydrodynamics analysis for an upflow integrated anaerobic digestion reactor with microbial electrolysis under different hydraulic retention times: effect of bioelectrode spatial distribution on functional communities involved in methane production and organic removal. ACS Sustain Chem Eng.

[CR31] Garedew M, Lin F, Song B (2020). Greener routes to biomass waste valorization: lignin transformation through electrocatalysis for renewable chemicals and fuels production. Chemsuschem.

[CR32] Gou C, Yang Z, Huang J (2014). Effects of temperature and organic loading rate on the performance and microbial community of anaerobic co-digestion of waste activated sludge and food waste. Chemosphere.

[CR33] Gould JM (1984). High-efficiency ethanol production from lignocellulosic residues pretreated with alkaline H_2_O_2_. Biotechnol Bioeng.

[CR34] Guo P, Mochidzuki K, Cheng W (2011). Effects of different pretreatment strategies on corn stalk acidogenic fermentation using a microbial consortium. Bioresour Technol.

[CR35] Guo Z, Liu W, Yang C (2017). Computational and experimental analysis of organic degradation positively regulated by bioelectrochemistry in an anaerobic bioreactor system. Water Res.

[CR36] Holliger C, Alves M, Andrade D (2016). Towards a standardization of biomethane potential tests. Water Sci Technol.

[CR37] Hu Z, Wen Z (2008). Enhancing enzymatic digestibility of switchgrass by microwave-assisted alkali pretreatment. Biochem Eng J.

[CR38] Islam MdS, Guo C, Liu C-Z (2018). Enhanced hydrogen and volatile fatty acid production from sweet sorghum stalks by two-steps dark fermentation with dilute acid treatment in between. Int J Hydrog Energy.

[CR39] Janke L, Leite A, Batista K (2016). Optimization of hydrolysis and volatile fatty acids production from sugarcane filter cake: effects of urea supplementation and sodium hydroxide pretreatment. Pretreat Biomass.

[CR40] Jankowska E, Chwiałkowska J, Stodolny M, Oleskowicz-Popiel P (2015). Effect of pH and retention time on volatile fatty acids production during mixed culture fermentation. Bioresour Technol.

[CR41] Jankowska E, Chwialkowska J, Stodolny M, Oleskowicz-Popiel P (2017). Volatile fatty acids production during mixed culture fermentation—the impact of substrate complexity and pH. Chem Eng J.

[CR42] Jiang J, Zhang Y, Li K (2013). Volatile fatty acids production from food waste: effects of pH, temperature, and organic loading rate. Bioresour Technol.

[CR43] Jiang Y, May HD, Lu L (2019). Carbon dioxide and organic waste valorization by microbial electrosynthesis and electro-fermentation. Water Res.

[CR44] Jiang Y, Chu N, Zhang W (2020). Electro-fermentation regulates mixed culture chain elongation with fresh and acclimated cathode. Energy Convers Manag.

[CR45] Kainthola J, Kalamdhad AS, Goud VV, Goel R (2019). Fungal pretreatment and associated kinetics of rice straw hydrolysis to accelerate methane yield from anaerobic digestion. Bioresour Technol.

[CR46] Kandylis P, Bekatorou A, Pissaridi K (2016). Acidogenesis of cellulosic hydrolysates for new generation biofuels. Biomass Bioenergy.

[CR47] Khan MA, Ngo HH, Guo W (2019). Selective production of volatile fatty acids at different pH in an anaerobic membrane bioreactor. Bioresour Technol.

[CR48] Khanal SK, Chen W-H, Li L, Sung S (2004). Biological hydrogen production: effects of pH and intermediate products. Int J Hydrog Energy.

[CR49] Kim N-J, Park GW, Kang J (2013). Volatile fatty acid production from lignocellulosic biomass by lime pretreatment and its applications to industrial biotechnology. Biotechnol Bioprocess Eng.

[CR50] Kim JS, Lee YY, Kim TH (2016). A review on alkaline pretreatment technology for bioconversion of lignocellulosic biomass. Pretreat Biomass.

[CR51] Kocher GS, Kaur P, Taggar MS (2017). An overview of pretreatment processes with special reference to biological pretreatment for rice straw delignification. Curr Biochem Eng.

[CR52] Kumar G, Mudhoo A, Sivagurunathan P (2016). Recent insights into the cell immobilization technology applied for dark fermentative hydrogen production. Bioresour Technol.

[CR53] Lansing S, Hülsemann B, Choudhury A (2019). Food waste co-digestion in Germany and the United States: from lab to full-scale systems. Resour Conserv Recycl.

[CR54] Latif MA, Mehta CM, Batstone DJ (2017). Influence of low pH on continuous anaerobic digestion of waste activated sludge. Water Res.

[CR55] Lee WS, Chua ASM, Yeoh HK, Ngoh GC (2014). A review of the production and applications of waste-derived volatile fatty acids. Chem Eng J.

[CR56] Li X, Chen H, Hu L (2011). Pilot-scale waste activated sludge alkaline fermentation, fermentation liquid separation, and application of fermentation liquid to improve biological nutrient removal. Environ Sci Technol.

[CR57] Li D, Liu S, Mi L (2015). Effects of feedstock ratio and organic loading rate on the anaerobic mesophilic co-digestion of rice straw and cow manure. Bioresour Technol.

[CR58] Liu X, Liu H, Chen Y (2008). Effects of organic matter and initial carbon–nitrogen ratio on the bioconversion of volatile fatty acids from sewage sludge. J Chem Technol Biotechnol.

[CR59] Liu H, Wang J, Wang A, Chen J (2011). Chemical inhibitors of methanogenesis and putative applications. Appl Microbiol Biotechnol.

[CR60] Liu H, Wang J, Liu X (2012). Acidogenic fermentation of proteinaceous sewage sludge: effect of pH. Water Res.

[CR61] Liu S, Bischoff KM, Leathers TD (2013). Butyric acid from anaerobic fermentation of lignocellulosic biomass hydrolysates by *Clostridium tyrobutyricum* strain RPT-4213. Bioresour Technol.

[CR62] Liu H, Wang Y, Yin B (2016). Improving volatile fatty acid yield from sludge anaerobic fermentation through self-forming dynamic membrane separation. Bioresour Technol.

[CR63] Liu J, Yu D, Zhang J (2016). Rheological properties of sewage sludge during enhanced anaerobic digestion with microwave-H_2_O_2_ pretreatment. Water Res.

[CR64] Liu H, Han P, Liu H (2018). Full-scale production of VFAs from sewage sludge by anaerobic alkaline fermentation to improve biological nutrients removal in domestic wastewater. Bioresour Technol.

[CR65] Liu L, Zhang Z, Wang J (2019). Simultaneous saccharification and co-fermentation of corn stover pretreated by H_2_O_2_ oxidative degradation for ethanol production. Energy.

[CR66] Liu S, Deng Z, Li H, Feng K (2019). Contribution of electrodes and electric current to process stability and methane production during the electro-fermentation of food waste. Bioresour Technol.

[CR67] Liu T, Zhou X, Li Z (2019). Effects of liquid digestate pretreatment on biogas production for anaerobic digestion of wheat straw. Bioresour Technol.

[CR68] Liu J, Yin J, He X (2021). Optimizing food waste hydrothermal parameters to reduce Maillard reaction and increase volatile fatty acid production. J Environ Sci.

[CR69] Lu X, Wang H, Ma F (2018). Improved process performance of the acidification phase in a two-stage anaerobic digestion of complex organic waste: effects of an iron oxide-zeolite additive. Bioresour Technol.

[CR70] Macias-Corral M, Samani Z, Hanson A (2008). Anaerobic digestion of municipal solid waste and agricultural waste and the effect of co-digestion with dairy cow manure. Bioresour Technol.

[CR71] Mao C, Feng Y, Wang X, Ren G (2015). Review on research achievements of biogas from anaerobic digestion. Renew Sustain Energy Rev.

[CR72] Martínez-Abad A, Giummarella N, Lawoko M, Vilaplana F (2018). Differences in extractability under subcritical water reveal interconnected hemicellulose and lignin recalcitrance in birch hardwoods. Green Chem.

[CR73] Mockaitis G, Bruant G, Guiot SR (2020). Acidic and thermal pre-treatments for anaerobic digestion inoculum to improve hydrogen and volatile fatty acid production using xylose as the substrate. Renew Energy.

[CR74] Mohsenzadeh A, Jeihanipour A, Karimi K, Taherzadeh MJ (2012). Alkali pretreatment of softwood spruce and hardwood birch by NaOH/thiourea, NaOH/urea, NaOH/urea/thiourea, and NaOH/PEG to improve ethanol and biogas production. J Chem Technol Biotechnol.

[CR75] Monlau F, Barakat A, Steyer JP, Carrere H (2012). Comparison of seven types of thermo-chemical pretreatments on the structural features and anaerobic digestion of sunflower stalks. Bioresour Technol.

[CR76] Mu L, Zhang L, Zhu K (2020). Anaerobic co-digestion of sewage sludge, food waste and yard waste: synergistic enhancement on process stability and biogas production. Sci Total Environ.

[CR77] Murali N, Fernandez S, Ahring BK (2017). Fermentation of wet-exploded corn stover for the production of volatile fatty acids. Bioresour Technol.

[CR78] Musa M, Idrus S, Che Man H, Nik Daud N (2018). Wastewater treatment and biogas recovery using anaerobic membrane bioreactors (AnMBRs): strategies and achievements. Energies.

[CR79] Orfão JJM, Antunes FJA, Figueiredo JL (1999). Pyrolysis kinetics of lignocellulosic materials—three independent reactions model. Fuel.

[CR80] Pan X-R, Li W-W, Huang L (2018). Recovery of high-concentration volatile fatty acids from wastewater using an acidogenesis–electrodialysis integrated system. Bioresour Technol.

[CR81] Panigrahi S, Sharma HB, Dubey BK (2019). Overcoming yard waste recalcitrance through four different liquid hot water pretreatment techniques—structural evolution, biogas production and energy balance. Biomass Bioenergy.

[CR82] Park SK, Jang HM, Ha JH, Park JM (2014). Sequential sludge digestion after diverse pre-treatment conditions: sludge removal, methane production and microbial community changes. Bioresour Technol.

[CR83] Perendeci N, Gökgöl S, Orhon D (2018). Impact of alkaline H_2_O_2_ pretreatment on methane generation potential of greenhouse crop waste under anaerobic conditions. Molecules.

[CR84] Pu Y, Zhang D, Singh PM, Ragauskas AJ (2008). The new forestry biofuels sector. Biofuels Bioprod Biorefining.

[CR85] Puyuelo B, Gea T, Sánchez A (2010). A new control strategy for the composting process based on the oxygen uptake rate. Chem Eng J.

[CR86] Ragauskas AJ, Beckham GT, Biddy MJ (2014). Lignin valorization: improving lignin processing in the biorefinery. Science.

[CR87] Rago L, Pant D, Schievano A (2019). Electro-fermentation—microbial electrochemistry as new frontier in biomass refineries and industrial fermentations. Advanced bioprocessing for alternative fuels, biobased chemicals, and bioproducts.

[CR88] Ramos-Suarez M, Zhang Y, Outram V (2021). Current perspectives on acidogenic fermentation to produce volatile fatty acids from waste. Rev Environ Sci Biotechnol.

[CR89] Reddy KO, Maheswari CU, Shukla M, Rajulu AV (2012). Chemical composition and structural characterization of Napier grass fibers. Mater Lett.

[CR90] Regueiro L, Veiga P, Figueroa M (2012). Relationship between microbial activity and microbial community structure in six full-scale anaerobic digesters. Microbiol Res.

[CR91] Reilly M, Dinsdale R, Guwy A (2014). Mesophilic biohydrogen production from calcium hydroxide treated wheat straw. Int J Hydrog Energy.

[CR92] Ren N (1997). Ethanol-type fermentation from carbohydrate in high rate acidogenic reactor. Biotechnol Bioeng.

[CR93] Rouches E, Escudié R, Latrille E, Carrère H (2019). Solid-state anaerobic digestion of wheat straw: impact of S/I ratio and pilot-scale fungal pretreatment. Waste Manag.

[CR94] Saha BC, Yoshida T, Cotta MA, Sonomoto K (2013). Hydrothermal pretreatment and enzymatic saccharification of corn stover for efficient ethanol production. Ind Crops Prod.

[CR95] Saha M, Saynik PB, Borah A (2019). Dioxane-based extraction process for production of high quality lignin. Bioresour Technol Rep.

[CR96] Sanders ME, Klaenhammer TR (2001). Invited review: the scientific basis of *Lactobacillus acidophilus* NCFM functionality as a probiotic. J Dairy Sci.

[CR97] Saritpongteeraka K, Chaiprapat S, Boonsawang P, Sung S (2015). Solid state co-fermentation as pretreatment of lignocellulosic palm empty fruit bunch for organic acid recovery and fiber property improvement. Int Biodeterior Biodegrad.

[CR98] Sawatdeenarunat C, Sung S, Khanal SK (2017). Enhanced volatile fatty acids production during anaerobic digestion of lignocellulosic biomass via micro-oxygenation. Bioresour Technol.

[CR99] Seeliger S, Janssen PH, Schink B (2002). Energetics and kinetics of lactate fermentation to acetate and propionate via methylmalonyl-CoA or acrylyl-CoA. FEMS Microbiol Lett.

[CR100] Sharma HB, Panigrahi S, Dubey BK (2019). Hydrothermal carbonization of yard waste for solid bio-fuel production: study on combustion kinetic, energy properties, grindability and flowability of hydrochar. Waste Manag.

[CR101] Shi J, Wang Z, Stiverson JA (2013). Reactor performance and microbial community dynamics during solid-state anaerobic digestion of corn stover at mesophilic and thermophilic conditions. Bioresour Technol.

[CR102] Shi X, Lin J, Zuo J (2017). Effects of free ammonia on volatile fatty acid accumulation and process performance in the anaerobic digestion of two typical bio-wastes. J Environ Sci.

[CR103] Soltanian S, Aghbashlo M, Almasi F (2020). A critical review of the effects of pretreatment methods on the exergetic aspects of lignocellulosic biofuels. Energy Convers Manag.

[CR104] Song K, Chu Q, Hu J (2019). Two-stage alkali-oxygen pretreatment capable of improving biomass saccharification for bioethanol production and enabling lignin valorization via adsorbents for heavy metal ions under the biorefinery concept. Bioresour Technol.

[CR105] Song X, Wachemo AC, Zhang L (2019). Effect of hydrothermal pretreatment severity on the pretreatment characteristics and anaerobic digestion performance of corn stover. Bioresour Technol.

[CR106] Sturm-Richter K, Golitsch F, Sturm G (2015). Unbalanced fermentation of glycerol in *Escherichia coli* via heterologous production of an electron transport chain and electrode interaction in microbial electrochemical cells. Bioresour Technol.

[CR107] Sun C, Liu R, Cao W (2015). Impacts of alkaline hydrogen peroxide pretreatment on chemical composition and biochemical methane potential of agricultural crop stalks. Energy Fuels.

[CR108] Sun J, Li Z, Zhou X (2019). Investigation on methane yield of wheat husk anaerobic digestion and its enhancement effect by liquid digestate pretreatment. Anaerobe.

[CR109] Tahboub MB, Lindemann WC, Murray L (2008). Chemical and physical properties of soil amended with pecan wood chips. HortScience.

[CR110] Tao X, Zhang P, Zhang G (2019). Carbide slag pretreatment enhances volatile fatty acid production in anaerobic fermentation of four grass biomasses. Energy Convers Manag.

[CR111] Tezel U, Tandukar M, Pavlostathis SG, Moo-Young M (2011). 6.35—Anaerobic biotreatment of municipal sewage sludge. Comprehensive biotechnology (Second Edition).

[CR112] Tian D, Guo Y, Hu J (2020). Acidic deep eutectic solvents pretreatment for selective lignocellulosic biomass fractionation with enhanced cellulose reactivity. Int J Biol Macromol.

[CR113] Torri C, Samorì C, Ajao V (2019). Pertraction of volatile fatty acids through biodiesel-based liquid membranes. Chem Eng J.

[CR114] Tu W, Zhang D, Wang H (2019). Polyhydroxyalkanoates (PHA) production from fermented thermal-hydrolyzed sludge by mixed microbial cultures: the link between phosphorus and PHA yields. Waste Manag.

[CR115] Vidal BC, Dien BS, Ting KC, Singh V (2011). Influence of feedstock particle size on lignocellulose conversion—a review. Appl Biochem Biotechnol.

[CR116] Wainaina S, Lukitawesa, Kumar Awasthi M, Taherzadeh MJ (2019). Bioengineering of anaerobic digestion for volatile fatty acids, hydrogen or methane production: a critical review. Bioengineered.

[CR117] Wainaina S, Parchami M, Mahboubi A (2019). Food waste-derived volatile fatty acids platform using an immersed membrane bioreactor. Bioresour Technol.

[CR118] Wang X, Yang G, Feng Y (2012). Optimizing feeding composition and carbon-nitrogen ratios for improved methane yield during anaerobic co-digestion of dairy, chicken manure and wheat straw. Bioresour Technol.

[CR119] Wang D, Liu Y, Ngo HH (2017). Approach of describing dynamic production of volatile fatty acids from sludge alkaline fermentation. Bioresour Technol.

[CR120] Wang X, Li Z, Bai X (2018). Study on improving anaerobic co-digestion of cow manure and corn straw by fruit and vegetable waste: methane production and microbial community in CSTR process. Bioresour Technol.

[CR121] Wang S, Tao X, Zhang G (2019). Benefit of solid-liquid separation on volatile fatty acid production from grass clipping with ultrasound-calcium hydroxide pretreatment. Bioresour Technol.

[CR122] Wang X, Guo W, Wen Y (2019). Effects of temperature on lignocellulosic wastes hydrolysis and volatile fatty acids accumulation under neutral and strongly alkaline conditions. IOP Conf Ser Earth Environ Sci.

[CR123] Wikandari R, Taherzadeh MJ (2019). Rapid anaerobic digestion of organic solid residuals for biogas production using flocculating bacteria and membrane bioreactors—a critical review. Biofuels Bioprod Biorefin.

[CR124] World Bioenergy Association (2016) Global biomass potential towards 2035

[CR125] Wu Q, Bao X, Guo W (2019). Medium chain carboxylic acids production from waste biomass: current advances and perspectives. Biotechnol Adv.

[CR126] Xiang C, Tian D, Hu J (2021). Why can hydrothermally pretreating lignocellulose in low severities improve anaerobic digestion performances?. Sci Total Environ.

[CR127] Xu H, Li Y, Hua D (2021). Effect of microaerobic microbial pretreatment on anaerobic digestion of a lignocellulosic substrate under controlled pH conditions. Bioresour Technol.

[CR128] Yang L, Xu F, Ge X, Li Y (2015). Challenges and strategies for solid-state anaerobic digestion of lignocellulosic biomass. Renew Sustain Energy Rev.

[CR129] Yang Y, Yang J, Cao J, Wang Z (2018). Pretreatment with concurrent UV photocatalysis and alkaline H_2_O_2_ enhanced the enzymatic hydrolysis of sisal waste. Bioresour Technol.

[CR130] Yao Z, Li W, Kan X (2017). Anaerobic digestion and gasification hybrid system for potential energy recovery from yard waste and woody biomass. Energy.

[CR131] Yuan H, Song X, Guan R (2019). Effect of low severity hydrothermal pretreatment on anaerobic digestion performance of corn stover. Bioresour Technol.

[CR132] Zealand AM, Roskilly AP, Graham DW (2017). Effect of feeding frequency and organic loading rate on biomethane production in the anaerobic digestion of rice straw. Transform Innov Sustain Future Part II.

[CR133] Zhang Y, Hu J, Zhang Y, Cristol D (2018). Development of Chinese character-writing program for mobile devices. Handbook of mobile teaching and learning.

[CR134] Zhang B, Zhang L-L, Zhang S-C (2005). The influence of pH on hydrolysis and acidogenesis of kitchen wastes in two-phase anaerobic digestion. Environ Technol.

[CR135] Zhang X, Qiu W, Chen H (2012). Enhancing the hydrolysis and acidification of steam-exploded cornstalks by intermittent pH adjustment with an enriched microbial community. Bioresour Technol.

[CR136] Zhang J, Li W, Lee J (2017). Enhancement of biogas production in anaerobic co-digestion of food waste and waste activated sludge by biological co-pretreatment. Energy.

[CR137] Zhang L, Loh K-C, Zhang J (2018). Food waste enhanced anaerobic digestion of biologically pretreated yard waste: analysis of cellulose crystallinity and microbial communities. Waste Manag.

[CR138] Zhang L, Loh K-C, Zhang J (2019). Enhanced biogas production from anaerobic digestion of solid organic wastes: current status and prospects. Bioresour Technol Rep.

[CR139] Zhang L, Loh K-C, Kuroki A (2021). Microbial biodiesel production from industrial organic wastes by oleaginous microorganisms: current status and prospects. J Hazard Mater.

[CR140] Zhou M, Yan B, Wong JWC, Zhang Y (2018). Enhanced volatile fatty acids production from anaerobic fermentation of food waste: a mini-review focusing on acidogenic metabolic pathways. Bioresour Technol.

[CR141] Zhou M, Yan B, Lang Q, Zhang Y (2019). Elevated volatile fatty acids production through reuse of acidogenic off-gases during electro-fermentation. Sci Total Environ.

[CR142] Zhu Y, Yang S-T (2004). Effect of pH on metabolic pathway shift in fermentation of xylose by *Clostridium tyrobutyricum*. J Biotechnol.

[CR143] Zhu L, Li W, Dong X (2003). Species identification of genus *Bifidobacterium* based on partial HSP60 gene sequences and proposal of *Bifidobacterium thermacidophilum* subsp. porcinum subsp. nov. Int J Syst Evol Microbiol.

